# A booster hepatitis B vaccine for children with maternal HBsAg positivity before 2 years of age could effectively prevent vaccine breakthrough infections

**DOI:** 10.1186/s12879-022-07854-w

**Published:** 2022-11-18

**Authors:** Yarong Song, Xin Zhang, Minmin Liu, Xiangjun Zhai, Jianxun Liu, Yi Li, Lili Li, Yiwei Xiao, Zhongping Duan, Jing Jiang, Feng Ding, Liguo Zhu, Jie Jiang, Huaibin Zou, Hui Zhuang, Jie Wang, Jie Li

**Affiliations:** 1 Department of Microbiology & Infectious Disease Center, School of Basic Medical Sciences, Peking University Health Science Center, 38 Xueyuan Road, Haidian District, Beijing, 100083 China; 2 Jiangsu Provincial Center for Disease Control and Prevention, Nanjing, 210009 China; 3 Zhengzhou Municipal Center for Disease Control and Prevention, Zhengzhou, 450053 China; 4 Beijing Youan Hospital, Capital Medical University, Beijing, 100054 China; 5 Department of Clinical Research, First Hospital of Jilin University, Changchun, 130021 China

**Keywords:** Yeast recombinant hepatitis B vaccine, Long-term protective effect, Mothers with chronic HBV infection, Vaccine breakthrough infections, Booster

## Abstract

**Background:**

The long-term protective effect of hepatitis B vaccine (HepB), the incidence of hepatitis B virus (HBV) vaccine breakthrough infections (VBIs), and whether a booster HepB is necessary remain to be clarified in children born to mothers with chronic HBV infection.

**Methods:**

Based on a long-term follow-up prospective cohort of 1177 hepatitis B surface antigen (HBsAg)-positive mothers and their paired infants which was established from 2009 to 2011, total 454 children with immunoprophylaxis success as determined by postvaccination serologic testing (PVST) at 7 months old were included in this study. Among the 454 children, 246 never had a booster HepB, and 208 children received a booster HepB from 1 to 5 years of age. Multivariate logistic regression analysis was used to analyse the risk factors for HBV VBIs.

**Results:**

The hepatitis B surface antibody (anti-HBs) levels declined sharply from 7 months to 2 years old, and the anti-HBs seronegative rate in the children increased significantly from 2 years old. A total of 31 (6.83%) of the 454 children experienced VBIs, of which 7 had overt and 7 had occult HBV infections. Notably, 14 (45.16%) of the 31 children with VBIs were diagnosed at 2 years old, and all of them had anti-HBs positivity (> 10 mIU/mL) at 1 year old. Maternal hepatitis B e antigen (HBeAg) positivity, higher HBV DNA and HBsAg levels, lower initial infant anti-HBs levels and not receiving a booster HepB were independent risk factors for VBIs. The incidence of VBIs was significantly lower in children with a booster HepB than in nonboosted children (0.50 vs. 11.90%, *P* < 0.001), and none of the boosted children developed overt or occult HBV infection. The anti-HBs levels of 76.67% for the children with VBIs in the nonboosted group indicated positivity before VBIs was detected.

**Conclusions:**

After the primary full immunization with HepB, children born to mothers with chronic HBV infection, especially the children with maternal HBeAg positivity, high HBV DNA levels, high HBsAg levels and/or low initial infant anti-HBs levels, were at a high risk of VBIs, and a booster HepB for these children before 2 years old, instead of when their anti-HBs level is < 10 mIU/mL, could reduce the incidence of VBIs.

**Supplementary Information:**

The online version contains supplementary material available at 10.1186/s12879-022-07854-w.

## Background

Chronic hepatitis B (CHB), caused by hepatitis B virus (HBV) infection, is one of the most prevalent infectious diseases worldwide and poses a major public threat. Without antiviral treatment, CHB patients can progress to liver cirrhosis and hepatocellular carcinoma (HCC), which leads to approximately 820,000 deaths worldwide every year [1]. HBV is mainly transmitted through mother-to-child transmission (MTCT), blood and sexual contact. Whether HBV infection turns into chronic infection is closely related to the age of infection [2, 3]. The incidence of chronic HBV infection was 90% for newborns, 25–30% for children under 5 years old, and only 2–5% for the population over 5 years old post HBV infection [2]. Without any intervention, 70–90% of newborns who are positive for both maternal hepatitis B surface antigen (HBsAg) and hepatitis B e antigen (HBeAg), and 10–40% of newborns who are positive for maternal HBsAg alone will be chronically infected with HBV [4]. Hepatitis B vaccine (HepB) is the safest and most effective measure to prevent chronic HBV infection. Since HepB was adopted for neonatal universal immunization in the 1980s [5], the incidence of chronic HBV infection during the perinatal period and early childhood has been significantly reduced. However, the long-term protective effect of the vaccine has always been a concern; in particular, it is of great concern for children born to mothers with chronic HBV infection.

Previous studies have reported that the protective hepatitis B surface antibody (anti-HBs) levels induced by HepB gradually decrease over time but can persist for at least 15 years in the general population [6–8]. Even if the anti-HBs disappear, the memory, still present in immune cells, can also provide effective protection [9, 10]. However, some studies have shown that the immunological memory induced by HepB may disappear after a certain age rather than be maintained throughout life [11–13]. Since it is difficult for the protective effect of vaccines to reach 100%, the lower the protective effect is, the higher the risk of "vaccine breakthrough infections (VBIs)". In addition, individual differences may also lead to VBIs in individuals with low immune responses. It is well known that children younger than 5 years of age are usually in close contact with their mothers, which puts children born to chronic HBV-infected mothers at a high risk of HBV VBIs and subsequently makes it easy for them to develop chronic infection. Therefore, the long-term protective effect of HepB and the occurrence of VBIs in children born to chronic HBV-infected mothers are worthy of attention before the age of 5 years. It has been reported that 15–50% of children born to HBsAg-positive mothers had negative conversion of anti-HBs, 7–13% of the children born to HBeAg-positive mothers had VBIs, and 2–8% had HBsAg positivity 5 years after the primary full immunization with HepB. The incidence of VBIs was up to approximately 25% at 20 years after the primary full immunization with HepB [14–18]. Currently, children born to chronic HBV-infected mothers are managed according to the high-risk group of HBV infection; that is, their anti-HBs levels are monitored regularly, and a booster HepB should be given immediately, once their anti-HBs levels drop below the protection level (10 mIU/mL) [19–21]. However, there is a lack of exact real-world data on the efficacy of this measure.

In this study, 454 children were included to explore the dynamic changes of anti-HBs and the occurrence of VBIs to elucidate the protective effect of the yeast-recombinant HepB, which is the vaccine adopted by current immunization strategies. Therefore, this study might provide a scientific basis for formulating a better immunization strategy for children born to chronic HBV-infected mothers.

## Methods

### Patients

As previously reported, a long-term follow-up prospective cohort of 1177 HBsAg-positive mothers and their paired infants was established by our group from 2009 to 2011 [22], in which all infants received a 10 μg/dose of the recombinant yeast-derived HepB at birth (within 12 h), 1 month (± 7 d) and 6 (± 7 d) months old. The mothers enrolled in each centre were numbered in accordance with the order of delivery; the neonates born to mothers coded with odd numbers were treated with 100 IU hepatitis B immunoglobulin (HBIG), and the neonates born to mothers coded with even numbers were treated with 200 IU HBIG at birth (within 12 h). All infants were suggested to return for postvaccination serologic testing (PVST) at 7 months of age and to return annually at the ages of 1, 2, 3 and 5 years. A booster HepB was recommended for children with anti-HBs levels < 10 mIU/ml during follow-up.

Among the 1177 infants enrolled in our prospective cohort study, 1138 (96.69%) infants had immunoprophylaxis success at 7 months of age, of which 454 children participated in the subsequent annual follow-up visit until 5 years of age and were enrolled in this study (Additional file 1: Fig. S1); their mothers were also included. To exclude the bias of the lost follow-up population, the baseline characteristics between the 454 children and those lost to follow-up were compared, and there was no difference in the baseline characteristics between the two groups (Additional file 1: Table S1). Among the 454 children, 246 (54.19%) children never had a booster HepB, and 208 (45.81%) children received a booster HepB from 1 to 5 years of age. Specifically, 19 (4.19%), 109 (24.01%), and 80 (17.62%) children received a booster HepB at 1–2, 2–3 and 3–5 years of age, respectively. This study was approved by the Ethics Committee of Peking University Health Science Center (IRB00001052-12041 and IRB00001052-12042). Written informed consent forms were obtained from each mother and also obtained from the parents/guardians of the minors included in this study.

### HBV serological assays

HBV serological markers, including HBsAg, HBeAg, anti-HBs, hepatitis B e antibody (anti-HBe) and hepatitis B core antibody (anti-HBc) levels, were determined by an Abbott Architect i2000SR analyser (Abbott Diagnostic, Chicago, IL, USA) based on chemiluminescent microparticle immunoassay (CMIA). The detection range of the HBsAg assay was 0.05–250 IU/mL. Sera were manually diluted before further testing if HBsAg levels were higher than 250 IU/mL. Anti-HBs levels ≥ 10 mIU/mL were defined as positive. For HBeAg, anti-HBe and anti-HBc assays, the results were reported as the ratio of the relative light unit (RLU) to the cut off RLU (S/CO). An S/CO < 1.0 was considered negative for HBeAg and anti-HBc, and an S/CO > 1.0 was considered negative for anti-HBe.

### Quantitation of serum HBV DNA

Serum HBV DNA levels were quantitated using an Abbott Real-Time PCR Assay m2000 (m2000sp + m2000rt) system (Abbott Molecular, IL, USA). The lower (LLOD) and upper limits of detection were 1.18 log_10_ IU/mL (51 copies/mL) and 9.00 log_10_ IU/mL (3.41 × 10^9^ copies/mL), respectively. A level of HBV DNA less than the LLOD was reported as “not detected” or “ < 1.18 log_10_ IU/mL”, and an HBV DNA level ≥ 1.18 log_10_ IU/mL was considered positive.

### Polymerase chain reaction, direct sequencing and HBV genotyping

As previously described [23], HBV DNA was extracted by the Abbott m2000sp system, and then the S region of the HBV genome was amplified by PCR. The PCR products were sequenced, and the sequences were analysed by Mega 6.0 and BLAST (https://blast.ncbi.nlm.nih.gov/Blast.cgi). HBV genotyping was performed by a nPCR method previously established in our laboratory [23].

### Definitions

In this study, the PVST results for infants at 7 months of age were interpreted as follows: (1) immunoprophylaxis failure: HBsAg-positive, anti-HBs level < 10 mIU/mL; (2) non-response: HBsAg-negative, anti-HBs level < 10 mIU/mL; and (3) immunoprophylaxis success: HBsAg-negative, anti-HBs level ≥ 10 mIU/mL. Anti-HBs levels of 10–99.99, 100–999.99, and ≥ 1000 mIU/mL were defined as low, medium, and high levels, respectively [22].

When a child’s anti-HBs level was higher than that of the previous follow-up timepoint without any history of revaccination with HepB, it was likely to be a natural boosting, which was defined as either a twofold increase in the anti-HBs level when the previous anti-HBs level was ≥ 100 mIU/mL or a fourfold increase in the anti-HBs level when the previous anti-HBs level was 10–99.99 mIU/mL [24].

Since anti-HBc positivity in children before the age of 2 years is probably transmitted from maternal anti-HBc [16, 25], HBV VBIs were defined as anti-HBc positivity alone or combined with HBsAg and/or HBV DNA positivity from 2 years of age (inclusive). Furthermore, children who were positive for both anti-HBc and HBsAg were defined as having overt HBV infection, and children who were positive for both anti-HBc and HBV DNA were defined as having occult HBV infection (OBI).

### Statistical analysis

The statistical software package SPSS version 24.0 (SPSS, Chicago, IL) was used for all statistical analyses. Normally distributed data are expressed as the mean ± standard deviation and were compared by Student’s *t* test. Nonnormally distributed data are expressed as the median (range) and were compared by the Mann‒Whitney U test. Categorical variables are expressed as proportions (%, n/n) and were analysed by Chi-square tests/Fisher's exact test or the Kruskal‒Wallis H-test. Multivariate logistic regression analysis was used to calculate the odds ratio (OR) with a 95% confidence interval (CI) to analyse the risk factors for VBIs in children. The factors in the multivariate logistic regression models were selected from the factors with statistical significance in the univariate analysis between children with and without VBIs. In multivariate analysis, because multicollinearity would occur if highly correlated variables were used in the same model, maternal HBeAg status, HBV DNA levels and HBsAg levels were analysed separately in three models. All *P* values were two-tailed, and *P* < 0.05 was considered statistically significant.

## Results

### Comparison of the baseline characteristics of the children with or without a booster HepB

As shown in Table 1, among the 454 children, 246 (54.19%) children never received a booster HepB, and 208 (45.81%) children received a booster HepB from 1 to 5 years of age. The baseline characteristics of the children and their mothers were compared. Children with a booster HepB (booster group) had a lower initial anti-HBs level than those without a booster HepB (nonbooster group) (2.81 log_10_ mIU/mL *vs.* 2.93 log_10_ mIU/mL, *P* < 0.001), and there were no significant differences in the other baseline characteristics between the two groups. There was also no significant difference between the mothers of the two groups in prenatal baseline characteristics, including age, HBeAg positivity, HBV DNA levels, HBsAg levels and HBV genotypes.

**Table 1:** Comparison of the baseline characteristics of the children in the booster group and nonbooster group and those of their mothers

	Overall	Booster group	Nonbooster group	*P*^*c*^
Mothers				
Number	454	208	246	
Age (years), median (range)	25.70 (18.50–43.00)	25.85 (18.60–43.00)	25.65 (18.50–43.00)	0.825
HBeAg positivity, n (%)	150 (33.04%)	59 (28.37%)	91 (36.99%)	0.052
HBV DNA (log_10_ IU/mL), median (range)	3.03 (1.18–9.13)	2.95 (1.18–9.07)	3.14 (1.18–9.13)	0.150
HBsAg (log_10_ IU/mL), median (range)	3.59 (− 1.30–4.97)	3.62 (0.61–4.97)	3.56 (− 1.30–4.97)	0.913
Maternal genotype^a^, n				
B	38	18	20	0.935
C	265	116	149	
Mixed B + C, D	11	5	6	
Infants				
Sex, male: female	236: 218	107: 101	129:117	0.832
Anti-HBs (log_10_ mIU/mL), median (range)	2.88 (1.02–4.23)	2.81 (1.02–3.96)	2.93 (1.07–4.23)	< 0.001
Birth weight (kg), median (range)	3.50 (2.40–5.40)	3.40 (2.40–5.40)	3.50 (2.50–4.50)	0.086
Parturition manner, caesarean: vaginal	290: 164	130: 78	160: 86	0.574
Feeding pattern, breast^b^: artificial	163: 291	73: 135	90: 156	0.742

^a^Genotypes were not successfully identified for 140 pregnant women, including 69 and 71 pregnant women in the booster group and nonbooster group, respectively

^b^Breast feeding included mixed feeding

^c^*P *values represent the significant differences between the booster group and the nonbooster group

Furthermore, infants were divided into low (n = 38), medium (n = 247) and high (n = 169) response groups based on anti-HBs levels of 10–99.99, 100–999.99, and ≥ 1000 mIU/mL at 7 months of age, respectively. There were no significant differences in the baseline characteristics among the low-, medium- and high-response groups of infants and their paired mothers (Additional file 1: Table S2).

### The dynamic changes in anti-HBs levels in children after the primary full course of HepB vaccination

**Fig. 1:**
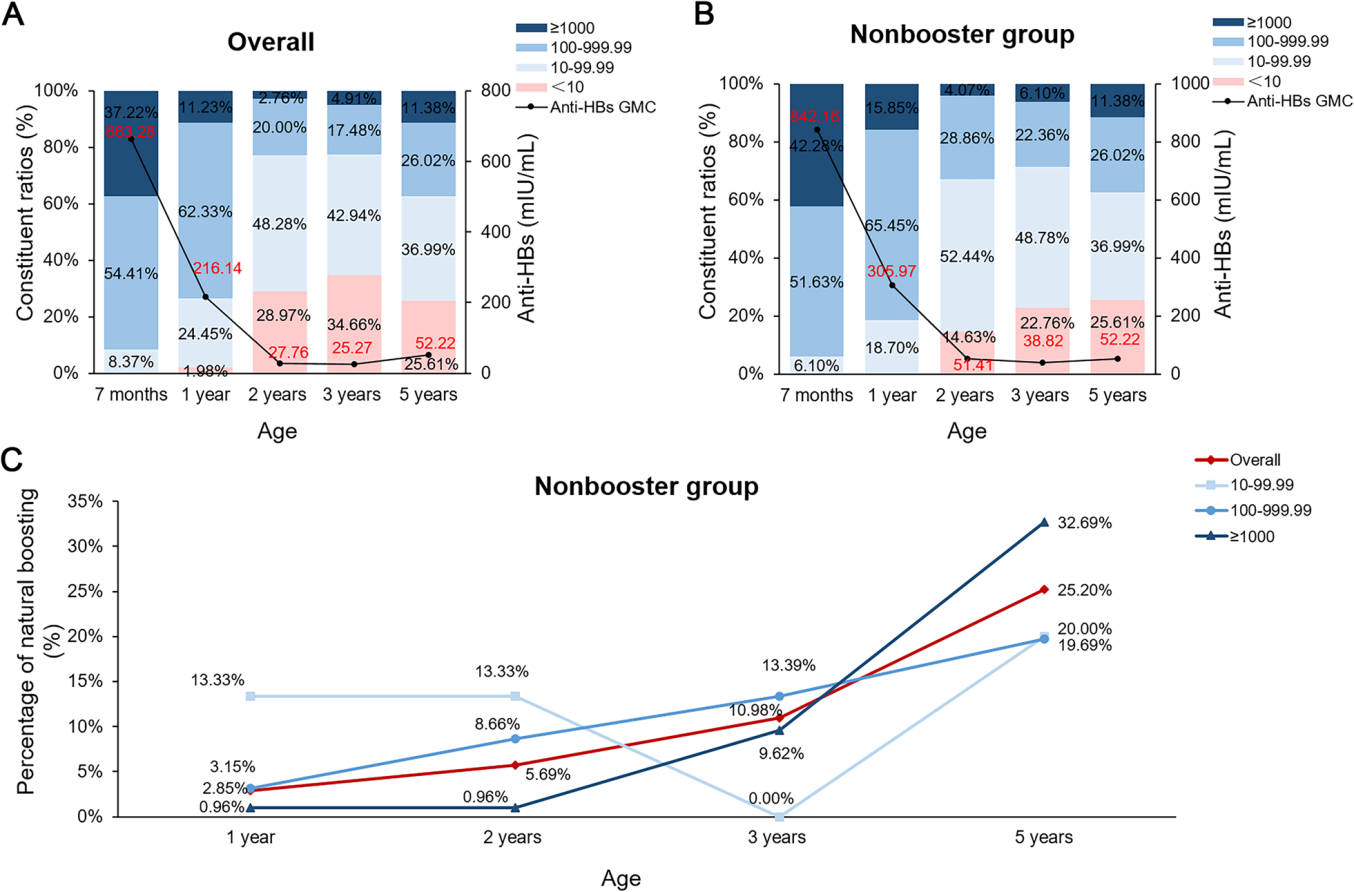
The dynamic changes in anti-HBs levels in children after primary full vaccination. (**A**) The constituent ratio and GMC of anti-HBs levels in all children. (**B**) The constituent ratio and GMC of anti-HBs levels in the nonbooster group. (**C**) The percentage of new natural boosting in the nonbooster group. Red numbers represent the line chart, and black numbers represent the bar chart. Anti-HBs, antibodies to HBsAg; GMC, geometric mean concentration

When calculating the dynamic change in anti-HBs levels, the children who received a booster HepB were excluded from the analysis of subsequent dynamic changes in anti-HBs levels. Among the 454 children followed up, 454, 454, 435, 326 and 246 children were included in the analysis of the dynamic changes in anti-HBs levels at 7 months, 1, 2, 3 and 5 years of age, respectively. As shown in Fig. 1A, the geometric mean concentrations (GMCs) of the anti-HBs levels were 663.28 (588.17–748.17), 216.14 (189.81–246.13), 27.76 (23.20–33.21), 25.27 (20.27–31.51) and 52.22 (38.59–70.68) mIU/mL, and the seronegative rates of anti-HBs levels were 0% (0/454), 1.98% (9/454), 28.97% (126/435), 34.66% (113/326) and 25.61% (63/246) at 7 months, 1, 2, 3 and 5 years of age, respectively. These results showed decreased levels of anti-HBs and increased seronegative rates of anti-HBs levels over time. Specifically, the GMC of anti-HBs levels declined sharply from 7 months to 2 years of age, and the seronegative rate of anti-HBs levels increased significantly from 2 years of age. Furthermore, the percentage of low anti-HBs levels increased, and the percentage of medium and high anti-HBs levels decreased significantly from 2 years of age. These results suggested that the protective efficacy of anti-HBs levels induced by HepB was greatly reduced from 2 years of age.

Among the 246 nonboosted children, the GMCs of the anti-HBs levels were 842.16 (716.83–989.41), 305.97 (261.02–358.66), 51.41 (41.17–64.19), 38.82 (30.23–49.85) and 52.22 (38.59–70.68) mIU/mL, and the seronegative rates of the anti-HBs levels were 0% (0/246), 0% (0/246), 14.63% (36/246), 22.76% (56/246) and 25.61% (63/246) at 7 months, 1, 2, 3 and 5 years of age, respectively (Fig. 1B). Consistent with the above results, the GMC of the anti-HBs levels declined sharply from 7 months to 2 years of age, the seronegative rate of anti-HBs levels and the percentage of low anti-HBs levels increased, and the percentage of medium and high anti-HBs levels decreased significantly from 2 years of age.

This result indicated that the children experienced a natural boosting of HBV when their anti-HBs level was higher than that of the previous follow-up timepoint without any history of revaccination for HepB, and they were at a high risk of VBIs. A total of 98 of the 246 (39.84%) children experienced natural boosting in the nonbooster group, and 12 of them experienced more than one boosting, which indicated that these children were at a high risk of HBV exposure. As shown in Fig. 1C, natural boosting occurred in 2.85% (7/246), 5.69% (14/246), 10.98% (27/246) and 25.20% (62/246) of the children at 1, 2, 3 and 5 years of age, respectively.

### The occurrence of VBIs

Since anti-HBc positivity in children before the age of 2 is probably transmitted from maternal anti-HBc [16, 25], HBV VBIs were defined as anti-HBc positivity alone or combined with HBsAg and/or HBV DNA positivity after 2 years of age. Furthermore, children with HBsAg positivity were defined as having an overt HBV infection, and children with HBsAg negativity but HBV DNA positivity were defined as having OBI. As shown in Table 2, 31 (6.83%) of the 454 children experienced VBIs, of which 7 (1.54%) had overt HBV infection and 7 (1.54%) had OBI. There were 14 (3.08%), 3 (0.66%) and 14 (3.08%) children with VBIs at 2, 3 and 5 years of age, respectively. Notably, among the children with VBIs, 45.16% (14/31) were diagnosed at 2 years of age, of whom 50.00% (7/14) had OBI. In particular, 7 (50.00%) of the 14 children with VBIs at 5 years of age had overt HBV infection.

**Table 2:** The incidence of VBIs among children with maternal HBsAg positivity during follow-up

Timepoint of VBIs detection	VBIs, n (%)	Type of VBIs		
		Anti-HBc ( +) alone, n (%)	Overt HBV infection, n (%)	Occult HBV infection, n (%)
Overall, (454)				
2 years of age	14 (3.08%)	7 (1.54%)	0	7 (1.54%)
3 years of age	3 (0.66%)	3 (0.66%)	0	0
5 years of age	14 (3.08%)	7 (1.54%)	7 (1.54%)	0
Total	31 (6.83%)	17 (3.74%)	7 (1.54%)	7 (1.54%)
Nonbooster group^a^, (252)				
2 years of age	14 (5.56%)	7 (2.78%)	0	7 (2.78%)
3 years of age	3 (1.19%)	3 (1.19%)	0	0
5 years of age	13 (5.16%)	6 (2.38%)	7 (2.78%)	0
Total	30 (11.90%)	16 (6.35%)	7 (2.78%)	7 (2.78%)
Booster group, (202)				
2 years of age	0	0	0	0
3 years of age	0	0	0	0
5 years of age	1 (0.50%)	1 (0.50%)	0	0
Total	1 (0.50%)	1 (0.50%)	0	0

^a^Six children who experienced HBV VBIs before receiving a booster HepB were included in the nonbooster group

Among children with VBIs, we randomly selected 4 paired mother–child samples with sufficient sera for PCR amplification and subsequent sequencing of the S region in the HBV genome, and the homology rate of the S gene sequences was compared between these children and their mothers. As shown in Additional file 1: Table S3, the homology rate of the HBV S region ranged from 97.94% to 100.00%, and the average genetic distance of the HBV strains was 0.022 (0.000–0.049) between the children and their mothers, which showed close homology and phylogenetic relationships for the mother–child pairs. Moreover, the common immune escape mutations in the "a" determinant were not found in the major hydrophilic domain region (MHR), whereas several novel mutations were found outside the major antigenic region.

### The risk factors associated with HBV VBIs

Together with those of their mothers, the baseline characteristics between children with and without VBIs were analysed. For the children with VBIs, the maternal HBeAg positive rate, HBV DNA and HBsAg levels were significantly higher than those of the children without VBIs. However, the initial infant anti-HBs levels for the children with VBIs were significantly lower than those of children without VBIs. There were no significant differences in the other baseline characteristics between the two groups (Additional file 1: Table S4).

Furthermore, a logistic regression analysis was performed on the factors related to HBV VBIs. As shown in Table 3, in univariate analysis, maternal HBeAg positivity (OR = 4.79, 95% CI: 2.19–10.45, *P* < 0.001), higher HBV DNA levels (OR = 1.31, 95% CI: 1.14–1.50, *P* < 0.001) and HBsAg levels (OR = 2.06, 95% CI: 1.23–3.45, *P* = 0.006), as well as lower levels of initial infant anti-HBs levels (OR = 0.43, 95% CI: 0.23–0.79, *P* = 0.006) and the absence of a booster HepB (OR = 27.16, 95% CI: 3.67–201.00, *P* = 0.001), were associated with a higher risk of VBIs in these children. Maternal HBeAg status, serum HBV DNA levels and HBsAg titres were positively correlated with each other [26, 27]. Therefore, in multivariate analysis, because multicollinearity would occur if highly correlated variables were used in the same model, maternal HBeAg status, HBV DNA levels and HBsAg levels were analysed separately in three models. Similarly, the results revealed that children with maternal HBeAg positivity (OR = 4.43, 95% CI: 1.94–10.12, *P* < 0.001), higher HBV DNA levels (OR = 1.30, 95% CI: 1.13–1.51, *P* < 0.001) and HBsAg levels (OR = 2.09, 95% CI: 1.20–3.62, *P* = 0.009), as well as lower initial infant anti-HBs titres (OR = 0.27, 95% CI: 0.13–0.55, *P* < 0.001) and the absence of a booster HepB (OR = 35.52, 95% CI: 4.65–271.57, *P* = 0.001) had a significantly higher risk of VBIs.

**Table 3:** Logistic regression analysis of the factors related to HBV VBIs

	Univariate		Multivariate^a^	
	OR (95% CI)	*P*	Adjusted OR (95% CI)	*P*
Mothers				
HBeAg (Positivity *vs*. Negativity)	4.79 (2.19–10.45)	< 0.001	4.43 (1.94–10.12)	< 0.001
HBV DNA level (per log_10_ IU/mL increase)	1.31 (1.14–1.50)	< 0.001	1.30 (1.13–1.51)	< 0.001
HBsAg level (per log_10_ IU/mL increase)	2.06 (1.23–3.45)	0.006	2.09 (1.20–3.62)	0.009
Age (per 1-year increase)	0.96 (0.88–1.03)	0.247		
HBV genotype (C *vs*. B)	1.05 (0.30–3.70)	0.937		
Infants				
Initial anti-HBs level (per log_10_ mIU/mL increase)	0.43 (0.23–0.79)	0.006	0.27 (0.13–0.55)	< 0.001
Parturition manner (Caesarean *vs*. Vaginal)	0.50 (0.24–1.04)	0.062		
Feeding pattern (Breast *vs*. Artificial)	1.28 (0.61–2.67)	0.521		
Booster HepB (no *vs*. yes)	27.16 (3.67–201.00)	0.001	35.52 (4.65–271.57)	0.001

^a^Maternal HBeAg status, HBV DNA levels and HBsAg levels were analysed separately in three models

According to the logistic regression analysis, the children were grouped by maternal HBeAg status, HBV DNA level, HBsAg level and initial infant anti-HBs level. The thresholds for maternal HBsAg status and HBV DNA levels were determined according to the thresholds of antiviral therapy of perinatal transmission [28]. As shown in Table 4, the children with maternal HBeAg positivity (14.00 vs. 3.29%, *P* < 0.001), HBV DNA levels ≥ 5.3 log_10_ IU/mL (14.18 vs. 3.75%, *P* < 0.001) or HBsAg levels ≥ 4 log_10_ IU/mL (11.76 vs. 4.72%, *P* = 0.006) had a higher incidence of VBIs than the children with maternal HBeAg negativity, HBV DNA levels < 5.3 log_10_ IU/mL or HBsAg levels < 4 log_10_ IU/mL. Based on the initial infant anti-HBs levels of the children at 7 months of age, we found that the lower the initial anti-HBs level was, the significantly higher the incidence of VBIs (15.79 vs. 8.10 vs. 2.96%, *P* = 0.004) (Table 5).

**Table 4:** The incidences of VBIs in different groups by maternal risk factors

Maternal factors	Overall	HBeAg status		*P*	HBV DNA (log_10_ IU/mL)		*P*	HBsAg (log_10_ IU/mL)		*P*
		Positivity	Negativity		< 5.3	≥ 5.3		< 4	≥ 4	
Overall										
Number	454	150	304		320	134		318	136	
VBIs, n (%)	31 (6.83)	21 (14.00)	10 (3.29)	< 0.001	12 (3.75)	19 (14.18)	< 0.001	15 (4.72)	16 (11.76)	0.006
Overt or occult HBV infection, n (%)	14 (3.08)	7 (4.67)	7 (2.30)	0.246	7 (2.19)	7 (5.22)	0.159	6 (1.89)	8 (5.88)	0.050
Nonbooster group										
Number	252	95	157		168	84		172	80	
VBIs, n (%)	30 (11.90)	20 (21.05)	10 (6.37)	< 0.001	12 (7.14)	18 (21.43)	0.001	15 (8.72)	15 (18.75)	0.022
Overt or occult HBV infection, n (%)	14 (5.56)	7 (7.37)	7 (4.46)	0.328	7 (4.17)	7 (8.33)	0.285	6 (3.49)	8 (10)	0.071
Booster group										
Number	202	55	147		152	50		146	56	
VBIs, n (%)	1 (0.50)	1 (1.82)	0	0.272	0	1 (2.00)	0.248	0	1 (1.79)	0.277
Overt or occult HBV infection, n (%)	0	0	0	-	0	0	-	0	0	-

**Table 5:** The incidences of VBIs in different groups by the initial anti-HBs levels of the children

Initial anti-HBs level (mIU/mL)	Overall	10–99.99	100–999.99	≥ 1000	*P*^*a*^
Overall					
Number	454	38	247	169	
VBIs, n (%)	31 (6.83%)	6 (15.79%)	20 (8.10%)	5 (2.96%)	0.004
Overt or occult HBV infection, n (%)	14 (3.08%)	2 (5.26%)	9 (3.64%)	3 (1.78%)	0.324
Nonbooster group					
Number	252	16	132	104	
VBIs, n (%)	30 (11.90%)	6 (37.50%)	19 (14.39%)	5 (4.95%)	< 0.001
Overt or occult HBV infection, n (%)	14 (5.56%)	2 (12.50%)	9 (6.82%)	3 (2.88%)	0.104
Booster group					
Number	202	22	115	65	
VBIs, n (%)	1 (0.50%)	1 (4.55%)	0	0	0.109
Overt or occult HBV infection, n (%)	0	0	0	0	–

^a^*P value*s represent the significant differences among three different distributions of anti-HBs titres

### The effect of a booster HepB on the occurrence of HBV VBIs

The children who did not receive a booster had a significantly higher risk of HBV VBIs. As shown in Table 2, among the nonbooster group, a total of 30 (11.90%, 30/252) children experienced VBIs, and the incidences of overt HBV infection and OBI were 2.78% (7/252) and 2.78% (7/252), respectively. There were 14 (5.56%), 3 (1.19%) and 13 (5.16%) children with VBIs at 2, 3 and 5 years of age, respectively. In the booster group, only 1 child experienced a VBI at 5 years of age, and this child was anti-HBc-positive. The incidences of VBIs (0.50 vs. 11.90%, *P* < 0.001) and overt or occult HBV infections (0 vs. 5.56%, *P* < 0.001) in the booster group were significantly lower than those in the nonbooster group, suggesting that a booster HepB could effectively prevent VBIs.

### The anti-HBs levels of the children at the follow-up timepoint prior to the detection of VBIs

**Fig. 2:**
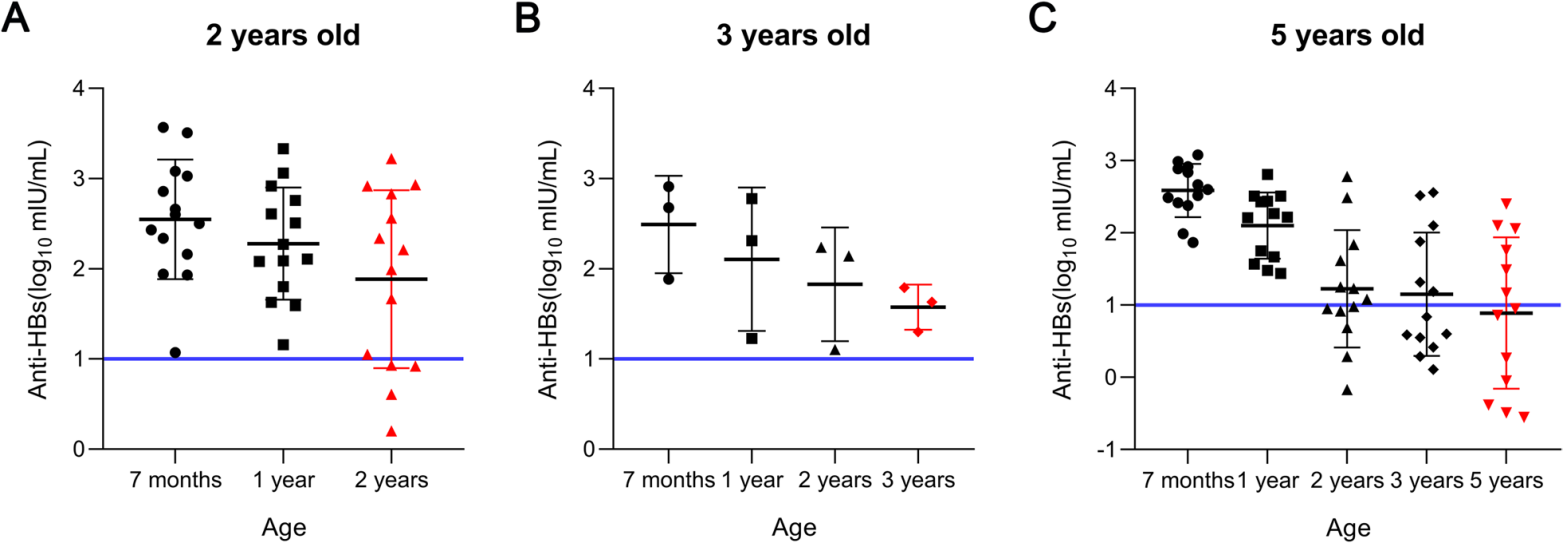
The anti-HBs levels at the previous timepoint of VBIs detection in the nonbooster group. (**A**) The anti-HBs level before VBIs detection at 2 years of age. (**B**) The anti-HBs level before VBIs detection at 3 years of age. (**C**) The anti-HBs level before VBIs detection at 5 years of age

In this study, all children were managed according to the high-risk population of HBV infection, and a booster HepB was recommended for those with anti-HBs levels < 10 mIU/ml during follow-up [19–21]. However, 23 (76.67%) of the 30 children who actually had VBIs in the nonbooster group had positive anti-HBs levels (> 10 mIU/mL) at the follow-up timepoint prior to VBIs detection (Fig. 2). Moreover, 14 (46.67%) of the 30 children with VBIs were diagnosed at the age of 2, and all 14 children had positive anti-HBs levels (> 10 mIU/mL) at 1 year of age.

## Discussion

It is of great concern when children are born to mothers with chronic HBV infection because these children are at a high risk of HBV infection due to close contact with their mothers until the age of 5. To date, there are still very few follow-up studies on the long-term protection of the recombinant HepB on newborns born to chronic HBV-infected mothers, especially regarding whether a booster dose is necessary for them [15, 16, 29, 30]. A systematic review summarized the immunogenicity of the recombinant HepB in children and showed that protective anti-HBs persisted in 68.4%-87.5% of the children born to HBsAg-positive mothers 5 years after primary full immunization with HepB [31]. In this study, the seronegative rate of anti-HBs levels increased by year, and the level of anti-HBs decreased gradually in the children born to chronic HBV-infected mothers. Specifically, the levels of anti-HBs declined sharply from 7 months of age [663.28 (588.17–748.17) mIU/mL] to 2 years of age [27.76 (23.20–33.21)) mIU/mL], and the seronegative rate of anti-HBs levels increased significantly from 2 years of age, especially in children with low initial levels of anti-HBs, which might not be enough to protect these children from VBIs when HBV attacks them due to their immature immune system. Moreover, 39.84% of the children who did not receive a booster HepB experienced natural boosting, and more than half of them were 5 years old, which led to a slight increase in the level of anti-HBs at 5 years of age. These results indicated that a considerable proportion of the children born to chronic HBV-infected mothers were susceptible to HBV infection.

Positive anti-HBc levels in children before the age of 2 mainly result from the passive transfer of maternal anti-HBc; thus, a positive anti-HBc level from 2 years of age may be regarded as an HBV VBI [16, 25]. Several reports showed that 7%-13% of the children born to HBeAg-positive mothers experienced VBIs within 5 years after the primary full immunization with the recombinant HepB [15–17]. The clinical consequence of OBI has been reported in the context of blood transfusion, liver transplantation, immunosuppressive conditions, and HCC [32]. A previous study found that 70.37% (19/27) of HBsAg (−) and anti-HBc ( +) subjects had detectable serum HBV DNA [5]. Our results showed that the cumulative incidence of HBV VBIs in children born to mothers with chronic HBV infection was 6.83% (31/454), and 45.16% (14/31) of them had overt or occult HBV infection. Therefore, the children born to mothers with chronic HBV infection were at a high risk of VBIs, and a considerable proportion of children with VBIs were at a risk of developing chronic HBV infection.

Furthermore, we found that the children with maternal HBeAg positivity, higher HBV DNA levels, higher HBsAg levels and lower initial infant anti-HBs titres were at a high risk of HBV VBIs, and the incidence of VBIs in children with maternal HBeAg positivity (14.00 vs. 3.29%, *P* < 0.001), HBV DNA levels ≥ 5.3 log10 IU/mL (14.18 vs. 3.75%, *P* < 0.001) or HBsAg levels ≥ 4 log10 IU/mL (11.76 vs. 4.72%, *P* = 0.006) was higher than that in children with maternal HBeAg negativity, HBV DNA levels < 5.3 log10 IU/mL or HBsAg levels < 4 log10 IU/mL. Among children with low, medium, and high levels of initial anti-HBs, the lower the initial anti-HBs level was, the significantly higher the incidence of VBIs (15.79 vs. 8.10 vs. 2.96%, *P* = 0.004). Therefore, sufficiently high levels of anti-HBs are needed to provide effective protection for these children who are frequently exposed to HBV.

It has been reported that maternal HBsAg positivity was an independent risk factor for developing VBIs, and a booster HepB could decrease HBV infection [33]. Consistently, our results indicated that the absence of a booster HepB was an independent risk factor for VBIs, and the incidences of VBIs (0.50 vs. 11.90%, *P* < 0.001) and overt or occult HBV infection (0 vs. 5.56%, *P* < 0.001) in children with a booster HepB were significantly lower than those in children without a booster HepB, suggesting that a booster HepB could prevent the occurrence of VBIs. These data highlighted the need for a better HepB booster immunization strategy for children born to mothers with chronic HBV infection.

For people at a high risk of HBV infection, guidelines have recommended regularly monitoring their anti-HBs levels and revaccinating with a single dose of HepB when their anti-HBs level is lower than the protection level [19–21]. In this study, a booster HepB was recommended for children with anti-HBs levels < 10 mIU/mL during annual follow-up. However, it is worth noting that 76.67% of the children with VBIs had anti-HBs levels > 10 mIU/mL at the follow-up timepoint prior to VBIs detection, which revealed that the 1-year follow-up interval might be too long to grasp the period when the level of anti-HBs drops to < 10 mIU/mL, or the anti-HBs threshold level (> 10 mIU/mL) might be too low to protect children born to mothers with chronic HBV infection from HBV VBIs. For the follow-up interval, the frequency of follow-up may need to be increased, such as to two or three times a year; however, this would undoubtedly considerably increase the cost, and increasing the frequency of follow-up would further increase the rate of follow-up loss due to the high economic and time costs. In contrast, a dose of a booster HepB was more cost-effective due to its high cost-effectiveness. Regarding the anti-HBs threshold level, that is, an anti-HBs level > 10 mIU/mL might not be enough to protect children born to HBsAg-positive mothers from HBV VBIs, and these children are more likely to need a timely booster HepB to increase the level of anti-HBs. Otherwise, all 454 children included in this study were HBsAg-negative and anti-HBs positive at 7 months of age. Only 19 children had received a booster HepB before 2 years of age, and none of the 19 children experienced VBIs. However, among the other 435 children without a booster HepB before 2 years of age, 14 (3.22%, 14/435) had VBIs at 2 years of age, and it is worth noting that all 14 children with VBIs had an anti-HBs level of > 10 mIU/mL at 1 year of age. Therefore, it is better for children with maternal HBsAg positivity to receive a booster HepB before 2 years of age, such as at their return visit at the age of 1 year, instead of waiting until their anti-HBs levels turn negative.

To date, this is the first study to explore the incidence of VBIs in children with maternal HBsAg positivity through a mother-infant paired prospective annual follow-up study. It revealed the long-term protective effect of HepB, the occurrence of VBIs in children with maternal HBsAg positivity, and the necessity of a booster HepB for these children. Furthermore, this study showed that it is better to give a booster HepB to these children before 2 years of age instead of after their anti-HBs levels are reduced to < 10 mIU/mL, which would provide a scientific basis for formulating a more ideal immunization strategy for the children born to HBsAg-positive mothers. However, there were some limitations in this study. The HBV infection status of other family members, including the father, was not analysed, and the optimal time for a booster HepB in children needs to be further explored.

## Conclusions

In conclusion, after primary full immunization with HepB, children born to mothers with chronic HBV infection, especially children with maternal HBeAg positivity, high HBV DNA levels, high HBsAg levels and/or low initial infant anti-HBs levels, are at significantly high risk of HBV VBIs beginning at 2 years of age. Therefore, we recommend that a booster dose of HepB should be given to children born to mothers with chronic HBV infection before 2 years of age instead of waiting for the anti-HBs level to drop to < 10 mIU/mL.

## Supplementary Information

**Additional file 1: Fig. S1. **Enrolment and follow-up of participants. **Table S1. **Comparison of the baseline characteristics of children followed up and lost to follow-up and those of their mothers. **Table S2. **Comparison of baseline characteristics of children with different anti-HBs levels at the age of 7 months and those of their mothers. **Table S3. **Genotype, subtype, homology and S region mutation comparisons of infected mother-child HBV pairs. **Table S4. **Comparison of the baseline characteristics of children with and without HBV VBIs and those of their mothers.

## Data Availability

The datasets used during the current study are available from the corresponding author upon reasonable request.

## Abbreviations

Anti-HBc: Antibody to HBcAg
Anti-HBs: Antibody to HBsAg
CHB: Chronic hepatitis B
CMIA: Chemiluminescent microparticle immunoassay
GMC: Geometric mean concentration
HBeAg: Hepatitis B e antigen
HBIG: Hepatitis B immunoglobulin
HBsAg: Hepatitis B surface antigen
HBV: Hepatitis B virus
HCC: Hepatocellular carcinoma
HepB: Hepatitis B vaccine
LLOD: Lower limit of detection
MHR: Major hydrophilic domain region
MTCT: Mother-to-child transmission
OBI: Occult HBV infection
PVST: Postvaccination serologic testing
VBIs: Vaccine breakthrough infections
