# Supplementary material for: A booster hepatitis B vaccine for children with maternal HBsAg positivity before 2 years of age could effectively prevent vaccine breakthrough infections
Source: BMC Infect Dis. 2022 Nov 18;22:863. doi: 10.1186/s12879-022-07854-w (PMC9673382; doi:10.1186/s12879-022-07854-w)
Supplement: Supplementary file 1 — Additional file 1: Fig. S1. Enrolment and follow-up of participants. Table S1. Comparison of the baseline characteristics of children followed up and lost to follow-up and those of their mothers. Table S2. Comparison of baseline characteristics of children with different anti-HBs levels at the age of 7 months and those of their mothers. Table S3. Genotype, subtype, homology and S region mutation comparisons of infected mother-child HBV pairs. Table S4. Comparison of the baseline characteristics of children with and without HBV VBIs and those of their mothers. [file 12879_2022_7854_MOESM1_ESM.docx]

**A booster hepatitis B vaccine for children with maternal HBsAg positivity before 2 years of age could effectively prevent vaccine breakthrough infections**

**Authors:** Yarong Song^1^, Xin Zhang^1^, Minmin Liu^1^, Xiangjun Zhai^2^, Jianxun Liu^3^, Yi Li^1^, Lili Li^1^, Yiwei Xiao^1^, Zhongping Duan^4^, Jing Jiang^5^, Feng Ding^1^, Liguo Zhu^2^, Jie Jiang^2^, Huaibin Zou^4^, Hui Zhuang^1^*, Jie Wang^1^*and Jie Li^1^*


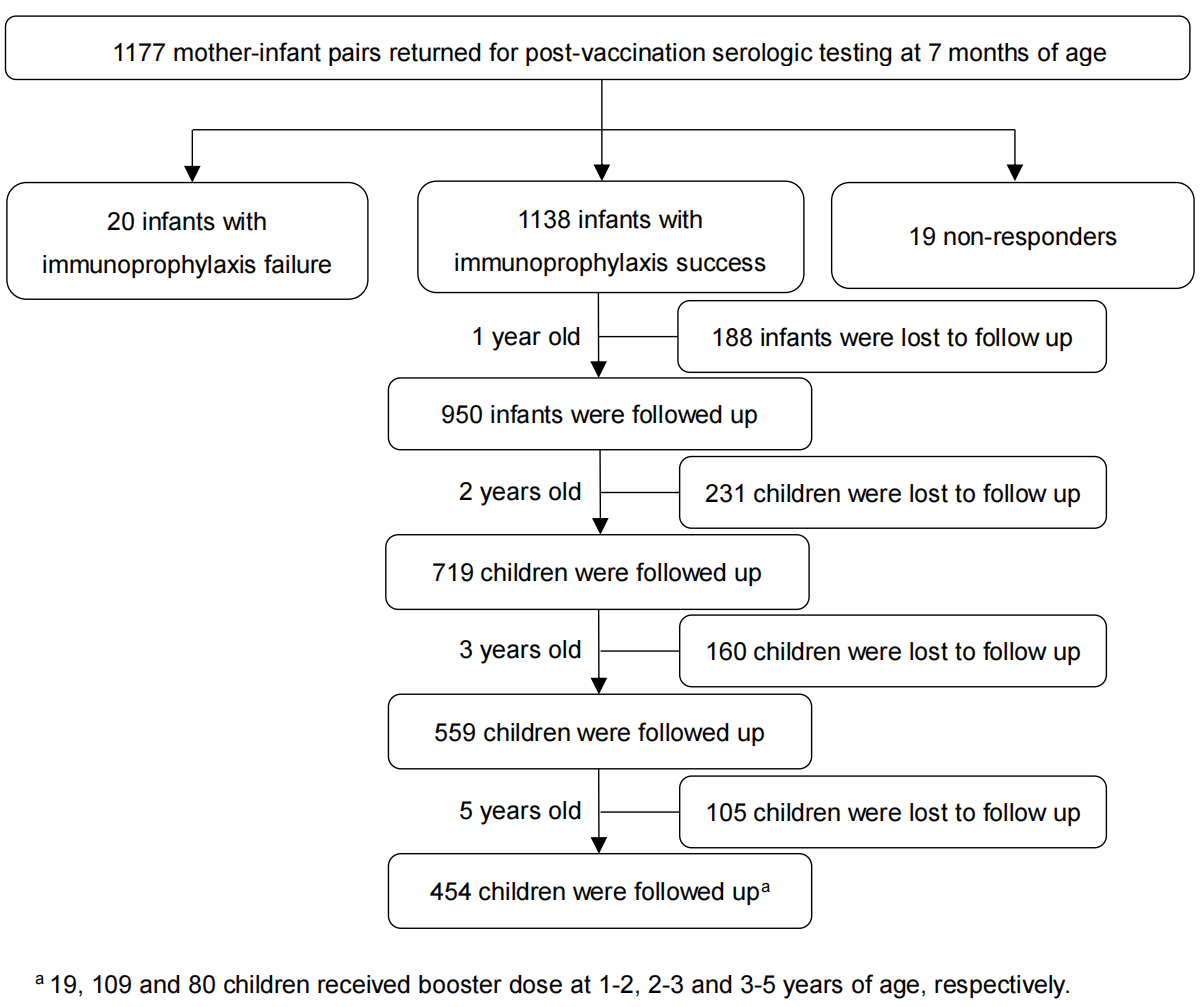


**Fig. S1** Enrollment and follow-up of participants.

**Table S1** Comparison of the baseline characteristics of children followed up and lost to follow-up and those of their mothers.

|  | | Overall | Continuous follow-up | Lost to follow-up | *P* |
| --- | --- | --- | --- | --- | --- |
| **Mother** | |  |  |  |  |
| Number | | 1138 | 454 | 684 |  |
| Age (years), median (range) | | 26.00 (15.10-43.00) | 25.70 (18.50-43.00) | 26.00 (15.10-42.60) | 0.674 |
| HBeAg positivity, n (%) | | 392 (34.45) | 150 (33.04) | 242 (35.38) | 0.416 |
| HBV DNA (log_10_ IU/mL), median(range) | | 3.25 (1.18-9.13) | 3.03 (1.18-9.13) | 3.27 (1.18-9.11) | 0.566 |
| HBsAg (log_10_ IU/mL), median(range) | | 3.61 (-1.30-5.03) | 3.59 (-1.30-4.97) | 3.61 (-0.92-5.03) | 0.742 |
| Maternal genotype^a^, n | B | 98 | 38 | 60 | 0.819 |
|  | C | 660 | 265 | 395 |  |
|  | Mixed B+C, D | 24 | 11 | 13 |  |
| **Infant** | |  |  |  |  |
| Gender, male: female | | 583:555 | 236: 218 | 347:337 |  |
| Anti-HBs (log_10_ mIU/mL) | | 2.89 (1.00-4.41) | 2.88 (1.02-4.23) | 2.89 (1.00-4.41) | 0.913 |
| Birth weight (kg), median (range) | | 3.50 (2.00-7.20) | 3.50 (2.40-5.40) | 3.45 (2.00-7.20) | 0.684 |

^a^Genotypes were not successfully identified in 356 pregnant women, including 140 and 216 pregnant women whose children were followed up and lost to follow-up, respectively.

**Table S2** Comparison of baseline characteristics of children with different anti-HBs levels at the age of 7 months and those of their mothers.

|  |  | Low response^a^ | Medium response | High response | *P* |
| --- | --- | --- | --- | --- | --- |
| **Mother** | |  |  |  |  |
| Number | | 38 | 247 | 169 |  |
| Age (years), median (range) | | 27.00 (19.70-35.00) | 25.00 (18.50-43.00) | 26.00 (19.00-40.00) | 0.719 |
| HBeAg positivity, n (%) | | 11 (28.95%) | 86 (34.82%) | 53 (31.36%) | 0.652 |
| HBV DNA (log_10_ IU/mL), median (range) | | 2.84 (1.18-8.72) | 3.09 (1.18-9.13) | 3.03 (1.18-9.06) | 0.713 |
| HBsAg (log_10_ IU/mL), median (range) | | 3.66 (0.61-4.90) | 3.59 (-0.62-4.97) | 3.57 (-1.30-4.88) | 0.846 |
| Maternal genotype^b^, n | B | 6 | 19 | 13 | 0.348 |
|  | C | 21 | 149 | 95 |  |
|  | Mixed B+C, D | 0 | 5 | 6 |  |
| **Infant** | |  |  |  |  |
| Gender, male: female | | 16: 22 | 132: 115 | 88: 81 | 0.428 |
| Birth weight (kg), median (range) | | 3.40 (2.50-4.20) | 3.50 (2.40-5.40) | 3.45 (2.50-4.70) | 0.721 |
| Parturition manner, cesarean: vaginal | | 25: 13 | 163: 84 | 102: 67 | 0.485 |
| Feeding pattern, breast^c^: artificial | | 12: 26 | 93: 154 | 58: 111 | 0.663 |

^a^The low, medium and high response groups represented that anti-HBs levels were 10-99.99, 100-999.99 and ≥1000 mIU/mL in infants at 7 months old.

^b^Genotypes were not successfully identified in 140 pregnant women, including 11, 74, and 55 pregnant women in low, medium and high response groups, respectively.

^c^Breast-feeding included mixed feeding.

**Table S3** Genotype, subtype, homology and S region mutation comparisons of infected mother-child HBV pairs.

| Mother-child pairs | Genotype | | Subtype | | Homology | S region Mutation | |
| --- | --- | --- | --- | --- | --- | --- | --- |
|  | Mother | Child | Mother | Child | S (%) | Mother | Child |
| E093 | C | C | adr | adr | 97.94 | N3S, I218L | I68T |
| E024 | C | C | adr | adr | 100 | V224A | V224A |
| E108 | C | C | adr | adr | 98.38 | S204R | None |
| TX157 | B | B | adw | adw | 98.24 | I4T, S59N | I4T, T45A, Q56P, C85F |

**Table S4** Comparison of the baseline characteristics of children with and without HBV VBIs and those of their mothers.

|  |  | Children with VBIs | Children without VBIs | *P* |
| --- | --- | --- | --- | --- |
| **Mother** | |  |  |  |
| Number | | 31 | 423 |  |
| Age (years), median (range) | | 24.00 (20.00-43.00) | 26.00 (18.50-43.00) | 0.186 |
| HBeAg positivity, n (%) | | 21 (67.74%) | 129 (30.50%) | <0.001 |
| HBsAg (log_10_ IU/mL), median (range) | | 4.01 (-0.62-4.89) | 3.55 (-1.30-4.97) | 0.001 |
| HBV DNA (log_10_ IU/mL), median (range) | | 7.89 (1.18-9.13) | 2.94 (1.18-9.07) | <0.001 |
| Maternal genotype^a^, n | B | 3 | 35 | 0.741 |
|  | C | 21 | 244 |  |
|  | Mixed B+C, D | 0 | 11 |  |
| **Infant** | |  |  |  |
| Gender, male: female | | 16:15 | 220: 203 | 0.966 |
| Anti-HBs (log_10_ mIU/mL), median (range) | | 2.60 (1.07-3.57) | 2.90 (1.02-4.23) | 0.003 |
| Birth weight (kg), median (range) | | 3.40 (2.75-4.10) | 3.50 (2.40-5.40) | 0.998 |
| Parturition manner, cesarean: vaginal | | 15: 16 | 275: 148 | 0.063 |
| Feeding pattern, breast^b^: artificial | | 11: 20 | 152: 271 | 0.960 |

^a^Genotypes were not successfully identified in 140 mothers, including 7 mothers whose children experienced HBV VBIs and 133 mothers whose children didn’t experience HBV VBIs.

^b^Breast-feeding included mixed feeding.
